# Radiographic findings in patients suspected of subacromial impingement syndrome: prevalence and reliability

**DOI:** 10.1007/s00256-024-04675-7

**Published:** 2024-04-23

**Authors:** Linda Christie Andrea, Susanne Wulff Svendsen, Poul Frost, Kate Smidt, John Gelineck, David Høyrup Christiansen, Søren Rasmussen Deutch, Torben Bæk Hansen, Jens Peder Haahr, Annett Dalbøge

**Affiliations:** 1 Present Address: Department of Radiology, Aarhus University Hospital, Aarhus, Denmark; 2 Department of Occupational Medicine– University Research Clinic - Danish Ramazzini Centre, Gødstrup Hospital, Herning, Denmark; 3 Danish Ramazzini Centre, Department of Occupational Medicine, Aarhus University Hospital, Aarhus, Denmark; 4 Department of Orthopedic Surgery, Viborg Regional Hospital, Viborg, Denmark; 5 Department of Radiology, Aarhus University Hospital, Aarhus, Denmark; 6 Present Address: Elective Surgery Centre, Silkeborg Regional Hospital, Silkeborg, Denmark; 7 Present Address: Department of Clinical Medicine, Aarhus University, Aarhus, Denmark; 8 Department of Orthopedic Surgery, Randers Regional Hospital, Randers, Denmark; 9 University Clinic of Hand, Hip and Knee Surgery, Holstebro Regional Hospital, Holstebro, Denmark

**Keywords:** Impingement, Radiograph, Shoulder, Reliability, Prevalence

## Abstract

**Objective:**

Aims were to (i) report prevalence and (ii) evaluate reliability of the radiographic findings in examinations of patients suspected of subacromial impingement syndrome (SIS), performed before a patient’s first consultation at orthopaedic department.

**Materials and methods:**

This cross-sectional study examined radiographs from 850 patients, age 18 to 63 years, referred to orthopaedic clinic on suspicion of SIS. Prevalence (%) of radiographic findings were registered. Inter- and intrarater reliability was analysed using expected and observed agreement (%), kappa coefficients, Bland–Altman plots, or intraclass coefficients.

**Results:**

A total of 850 patients with a mean age of 48.2 years (SD = 8.8) were included. Prevalence of the radiographic findings was as follows: calcification 24.4%, Bigliani type III (hooked) acromion 15.8%, lateral/medial acromial spurs 11.1%/6.6%, acromioclavicular osteoarthritis 12.0%, and Bankart/Hill-Sachs lesions 7.1%. Inter- and intrarater Kappa values for most radiographic findings ranged between 0.40 and 0.89; highest values for the presence of calcification (0.85 and 0.89) and acromion type (0.63 and 0.66). The inter- and intrarater intraclass coefficients ranged between 0.41 and 0.83; highest values for acromial tilt (0.79 and 0.83) and calcification area (0.69 and 0.81).

**Conclusion:**

Calcification, Bigliani type III (hooked) acromion, and acromioclavicular osteoarthritis were prevalent findings among patients seen in orthopaedic departments on suspicion of SIS. Spurs and Bankart/Hill-Sachs lesions were less common. Optimal reliabilities were found for the presence of calcification, calcification area, and acromial tilt. Calcification qualities, acromion type, lateral spur, and acromioclavicular osteoarthritis showed suboptimal reliabilities. Newer architectural measures (acromion index and lateral acromial angle) performed well with respect to reliability.

## Introduction

Subacromial impingement syndrome (SIS) or subacromial pain syndrome is a term used for a variety of shoulder disorders assumed to arise from subacromial pathologies [1, 2]. From 2007 to 2011, around 60,000 working age patients were diagnosed with SIS at Danish public and private hospitals [3]. Good practice according to Danish national clinical guidelines from 2013 comprise a routine radiographic examination before first evaluation at an orthopaedic department on suspicion of SIS [4]. Current recommendations of radiographic examinations comprise three projections (i.e. anterior–posterior (AP) views in external and internal rotation, and an outlet view). In patients evaluated for SIS, radiographic findings of presumed clinical importance include subacromial calcification [4–10], acromial morphological characteristics (i.e. hooked acromion or spurs) [4, 5, 7, 11–13], and signs of acromioclavicular osteoarthritis (OA) [5, 9, 11, 14, 15]. Furthermore, signs of previous trauma, including Bankart/Hill-Sachs lesion, which indicates previous luxation of the glenohumeral joint, are considered of clinical importance [16].

Knowledge of the prevalence of pathological findings in specific populations is necessary with respect to disease status. Previous studies have reported the prevalence of radiographic findings in e.g. the general population or specific occupational groups [17–20], primary care patients with shoulder pain [9, 14, 18], and patients diagnosed with SIS including rotator cuff tears [6, 7, 11, 13, 21–25]. We are not aware of studies of the prevalence of radiographic findings among patients referred to orthopaedic departments on suspicion of SIS.

The prevalence and clinical value of radiographic findings is, among other factors, influenced by the probability that a patient’s radiograph is rated likewise by different raters (interrater reliability) and—just as important—that individual raters report the same findings when re-evaluating a radiograph (intrarater reliability). However, reported inter- and (to a lesser degree) intrarater reliabilities have in general left much to be desired, e.g. poor to moderate reliabilities have been reported for calcification classifications [18, 26, 27], acromial type (Bigliani types I-III) [28–32], and presence/absence of acromial spurs [28]. We are not aware of studies that have examined whether individual calcification characteristics, such as density, show higher reliabilities than calcification classifications. Higher reliabilities have been reported for architectural measures, including acromial tilt (fair to good) [17, 33–35] and acromion index (excellent) [33], while we are not aware of reported reliabilities for lateral acromial angle [22].

The overall aim of this study was to describe the radiographic findings with reference to subacromial calcifications, acromial morphology, acromioclavicular OA, previous trauma, and architectural measures in orthopaedic patients examined on suspicion of SIS. The specific aims were to (i) report the prevalence of specific radiographic findings and (ii) to evaluate the inter- and intrarater reliability of the abovementioned radiographic findings. We hypothesised that (i) highest prevalence would be found for subacromial calcifications and acromioclavicular OA and (ii) reliability estimates > 0.5 could be achieved using a detailed manual to standardise the evaluations.

## Materials and methods

### Design and population

In this cross-sectional study, we used baseline information from a cohort study of shoulder patients in Central Denmark Region [36]. Included patients were registered in our project database, when they were referred from general practitioner to one of six public departments of orthopaedic surgery on suspicion of SIS in the period 1 January 2011 to 28 February 2012. Inclusion criteria was age 18 to 63 years, residence in 18 out of 19 municipalities in the Central Denmark Region (an island municipality was left out), and with at least one visit to department of orthopaedic surgery with suspected SIS. We excluded patients with no response to a questionnaire at first visit or before any surgery for SIS, and with no available radiograph of the shoulder from the relevant episode of care and prior to any surgery. To evaluate if our patient cohort were representative of all patients in Central Denmark Region, we obtained information from the Danish National Patient Register (DNPR) [37, 38] on all patients, who were registered with a visit to one of the relevant departments of orthopaedic surgery in the abovementioned period under a principal or secondary diagnosis in groups M75 (shoulder lesions) or M19.8 (other specified arthrosis) according to the International Classification of Diseases 10th revision, and who fulfilled the inclusion criteria with respect to age and residence.

The study was authorised by the Danish Data Protection Agency (journal number 2010–41-4316) and the Danish National Board of Health permitted the evaluation of radiographic examinations (reference number 3–3013-192/1/). In Denmark, questionnaire and register studies do not require approval by committees on health research ethics.

### Radiographic examination

**Fig. 1:**
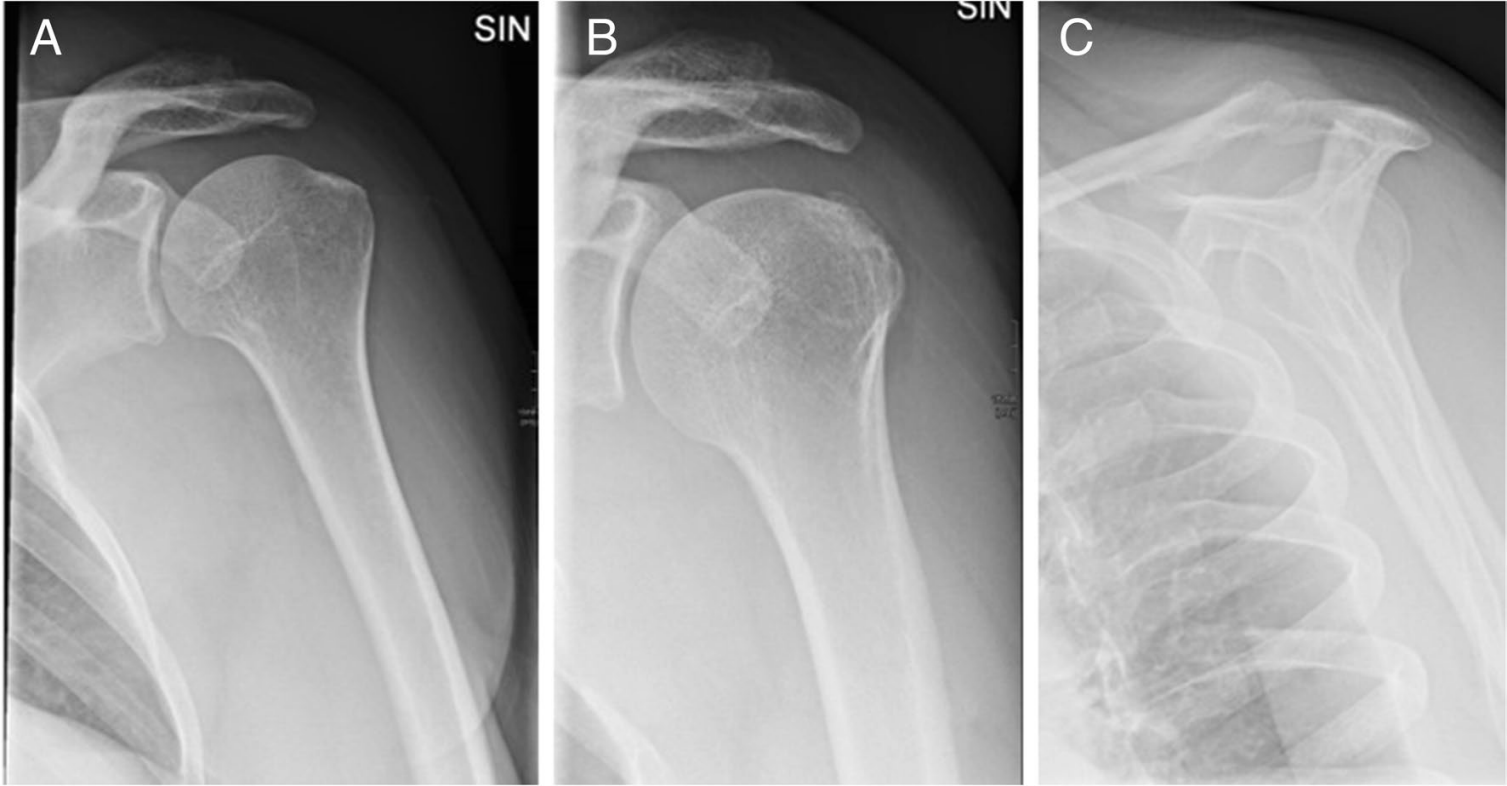
Standard projections. Anterior–posterior projections with humerus in external rotation (**A**) and internal rotation (**B**) and outlet view (**C**)

Radiographic examination was routinely performed before the first visit to a public department of orthopaedic surgery and the radiographs were digitally stored at regional servers. The radiographic examinations were performed by radiologic technologists, who were unaware of the study aim. The examination included up to three radiographs, i.e. AP views in external and internal rotation, and an outlet view (Fig. 1). In case of more than one examination, we selected the examination dated closest to the patient’s first visit to the public department of orthopaedic surgery. If this examination included both shoulders, we examined the radiographs of the right shoulder. We used IMPAX version 6.5 to evaluate the radiographs, including in-programme tools to measure calcification areas as well as angles and distances for architectural measures (see below). The radiographs were evaluated by two medical doctors at residential level of orthopaedic education (LCA and KS), supervised by an experienced musculoskeletal radiologist (JG). The evaluations were performed according to a detailed manual with extensive representation of illustrations, which we developed for the present study. To calibrate the evaluations initially, the evaluators individually evaluated 10 radiographs of SIS patients, who were not included in this study, and discussed any disagreements and doubts with the supervising radiologist. In accordance with clinical practice, the evaluators had information about the patients’ age and sex from patient data on the radiographs. Apart from the suspicion of SIS, neither clinical nor questionnaire information was available at the time of the evaluation.

**Fig. 2:**
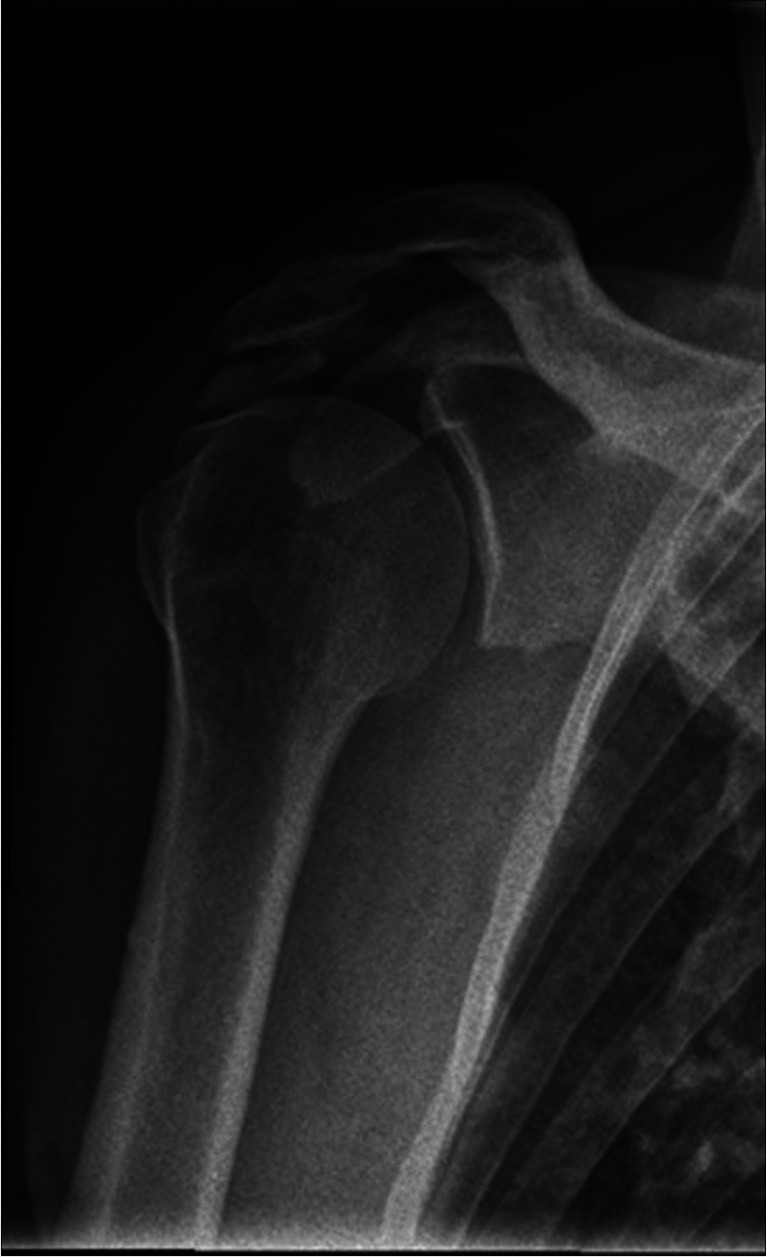
Subacromial calcification

**Fig. 3:**
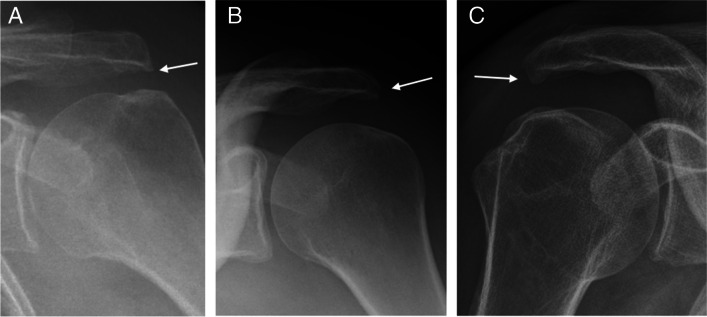
Lateral spurs in three different patients—bird beak type (**A** + **B**) and heel type (**C**)

**Fig. 4:**
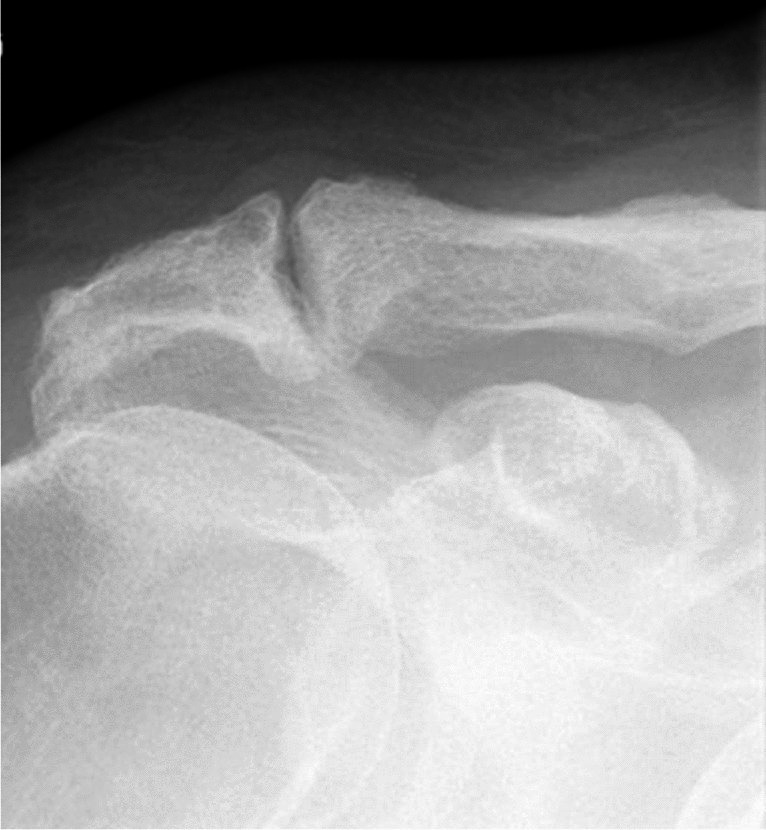
AC-joint arthritis with narrowing of joint and spurring of medial acromion

**Fig. 5:**
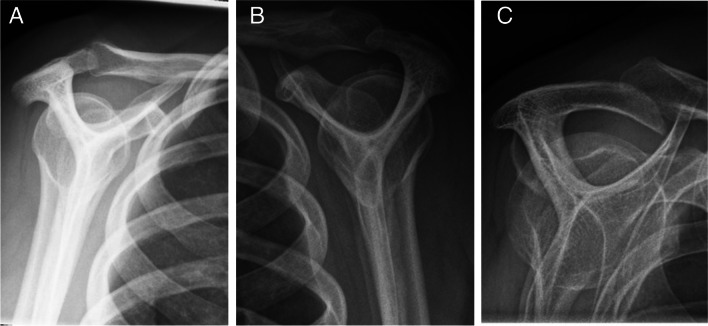
Acromion types. Type I—flat (**A**). Type II—curved (**B**). Type III—hooked (**C**)

**Fig. 6:**
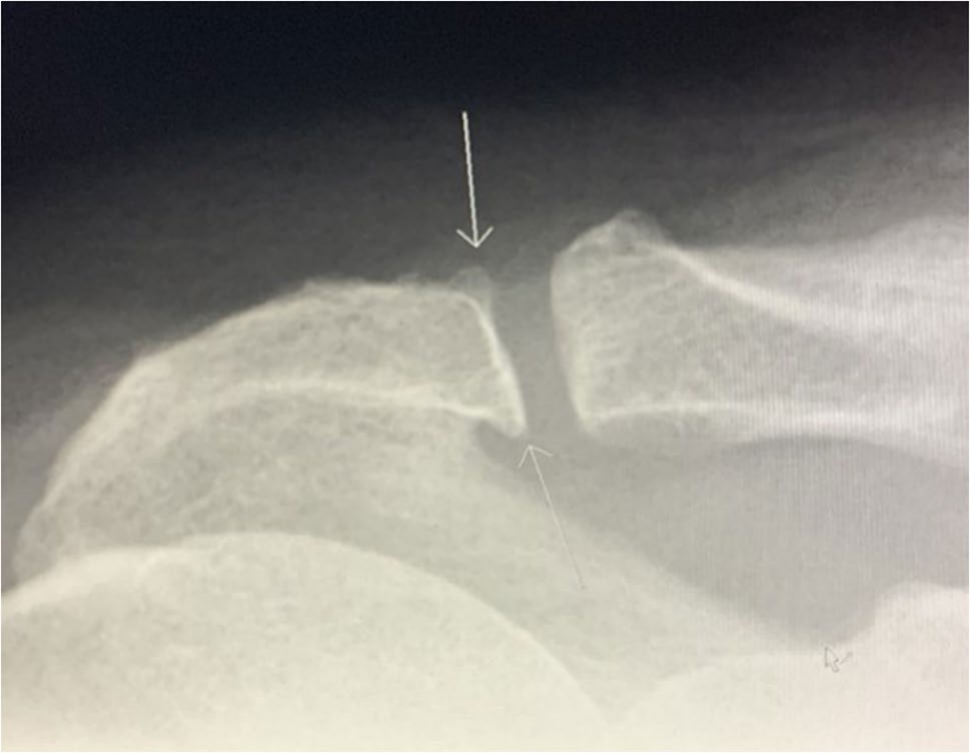
Medial acromion spurs

**Fig. 7:**
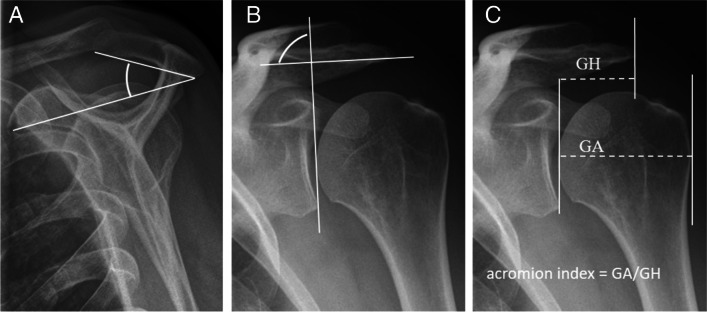
Architectural measures. Acromial tilt (**A**). Lateral acromial angle (**B**). Acromion index (**C**)

Radiographic findings based on evaluation of AP views comprised presence (no/yes), area, and characteristics of any calcifications (Fig. 2) (see below) ((of note, we disregarded calcifications that were only visible on outlet views (two patients)), presence (no/yes) and types of lateral acromial spurs (Fig. 3) [39], presence (no/yes) of acromioclavicular OA (Fig. 4) [40], and presence (no/yes) of Bankart/Hill-Sachs lesions [40]. Radiographic findings based on outlet views, comprised acromial type according to Bigliani (type I “flat”, type II “curved”, and type III “hooked”) (Fig. 5) [41, 42] and presence (no/yes) and types of medial acromial spurs (Fig. 6) [39]. The radiographic evaluation also included architectural measures in terms of acromial tilt (angle between undersurface of acromion and line from tip of coracoid process to posterior aspect of acromion), which was measured on outlet views [21], and acromion index (relationship between distance from glenoid fossa to lateral aspect of acromion acromion and the distance from the glenoid fossa to the lateral aspect of humerus) [43], and lateral acromial angle (angle between acromion undersurface and glenoid fossa) [22], which were measured on AP views (Fig. 7). An overview of these architectural measures has been provided previously [44].

We calculated calcification areas by multiplying the longest distance of the calcification by the longest perpendicular distance (cm^2^). In case of more than one calcification in an examination, we chose the one with the largest area for further characterisation of calcification qualities in terms of density (dense/transparent), homogeneity (homogeneous/inhomogeneous), circumscription (well-/ill-defined borders), dissemination (more than one calcification visible, no/yes), and localisation (insertion zone, no/yes). Calcification of rotator cuff components may represent calcific tendinopathy with deposition and subsequent resorption of calcium compounds in subacromial structures or degenerative/dystrophic calcifications, which are thought to be limited to the tendon insertion zone [45–47]. Based on the just-mentioned qualities, we classified the calcifications according to Gärtner [48], DePalma [49], Patte [50], and Molé [44] as presented in appendix 1. Calcifications in the insertion zone are included in Molé’s classification subclass D, whereas the remaining three systems seem to omit classification of calcifications in the insertion zone. The categories in the classifications of Patte, DePalma, and Molé are not exhaustive and mutually exclusive. Therefore, we applied decision algorithms: calcifications were classified as Patte type 2 if they did not meet the criteria for Patte type 1 and as DePalma type 1 if they did not meet the criteria for DePalma type 2. Regarding Molé’s classification, not all combinations of density, homogeneity, and circumscription have their own category (e.g. a calcification, which is rated inhomogeneous and ill-defined, does not fit into a specific classification category). To classify calcifications that did not fit into a specific category, we applied an algorithm based on density and homogeneity, disregarding circumscription.

### Inter- and intrarater reliability of radiographic findings

To evaluate the inter- and intrarater reliability, we randomly sampled one hundred radiographic examinations from each of two evaluators with an overrepresentation of calcifications so that the prevalence of calcifications seen by at least one evaluator was 40% (the evaluators were not informed about this percentage). The 200 examinations were re-evaluated independently by both evaluators, who were blinded in the sense that they did not know whether a given evaluation would be used to assess inter- or intrarater reliability. The time interval between the repeated evaluations was on average 55 weeks (range 10;110 weeks).

### Statistical analyses

We calculated the prevalence (%) of categorical radiographic findings, i.e. subacromial calcifications, acromial morphology, acromioclavicular osteoarthritis (OA), signs of previous trauma, and architectural measures. For continuous radiographic findings such as calcification area and architectural measures, we calculated mean and standard deviation (SD). Inter- and intrarater reliability of dichotomous outcomes was analysed in the subsample as expected and observed agreement (%) and kappa coefficients [51]. With 200 patients, for whom a positive finding is expected in 40%, a kappa coefficient of > 0.5 can be assessed with a 95% confidence interval (CI) of ± 0.12 (Stata sskdlg). For polytomous outcomes (i.e. acromial type, lateral spur type, and Moléʼs classification), we used kappa coefficients with quadratic weighting. The 95% CI for kappa values were obtained using nonparametric bootstrap methods (1000 replications)[52]. All kappa values were described according to Landis and Koch: < 0.00 “poor”, 0.00–0.20 “slight”, 0.21–0.40 “fair”, 0.41–0.60 “moderate”, 0.61–0.80 “substantial”, and 0.81–1.00 “almost perfect” [53]. For continuous outcomes (i.e. acromial tilt, acromion index, lateral acromial angle, and calcification area), we used Bland–Altman plots with 95% limits of agreement. Because the difference between the two ratings of calcification area was related to the mean of the rated areas we log-transformed this variable [54]. For the continuous outcomes, we also calculated intraclass coefficients (ICCagreement) from two-way random effects models as measures of reliability [55]. We described ICCagreement according to Fleiss et al.: < 0.40 “poor”, 0.40–0.75 “fair to good”, and > 0.75 “excellent” [56]. Reliability results were reported according to published guidelines [57].

## Results

**Fig. 8:**
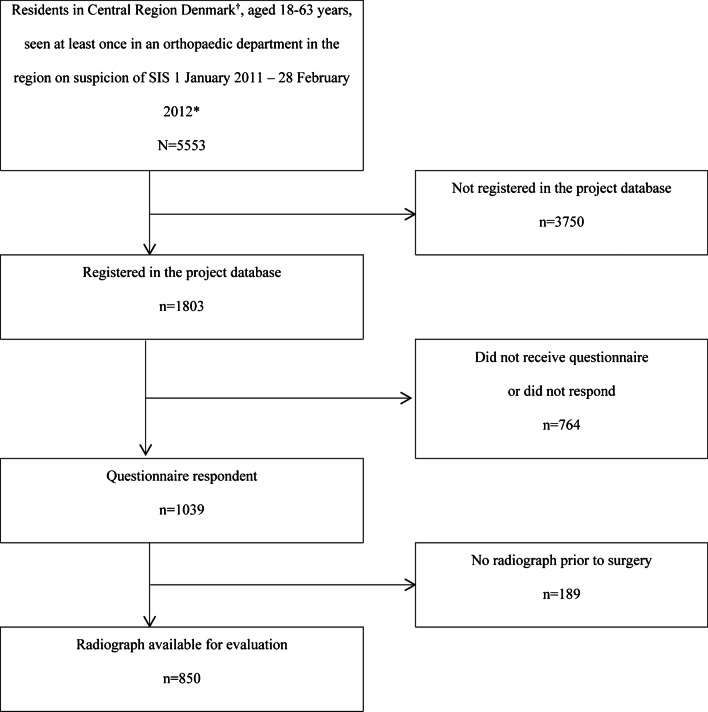
Flowchart of the study

Figure 8 presents the flow chart of the study population. Overall, we received a questionnaire from 57.6% (1039/1803) of the patients, who were registered in the project database. This percentage varied between 40.5% (201/496) and 60.8% (141/232) for the six participating departments. Among the questionnaire respondents, 81.8% (850/1039) had at least one available radiograph of the shoulder. Thus, the study comprised 850 patients, of whom 53.8% (457/850) were female. The mean age was 48.2 years (SD = 8.8). When we compared the 850 participants with the 5553 patients registered in the Danish National Patient Register, almost similar distribution of sex, age, and orthopaedic department was found (results not shown). Table 1 presents an overview of the available examinations for the 850 included patients. Only 24.5% (208/850) of the examinations comprised all three recommended projections, while 73.6% (626/850) comprised two projections.

**Table 1:** Available radiographic projections of the shoulder among patients referred to orthopaedic departments on suspicion of subacromial impingement (*n* = 850)

Available projections	*N*	%
AP internal and external rotation + outlet view	208	24.5
AP internal rotation + outlet view	252	29.6
AP external rotation + outlet view	334	39.3
AP internal and external rotation	40	4.7
AP internal rotation	6	0.7
AP external rotation	5	0.6
Outlet view	5	0.6

Table 2 presents the overall prevalence of the radiographic findings. The prevalence of calcification was 24.4% (207/850) of which 66.7% (138/207) were located in the insertion zone. We found a mean calcification area of 0.74 cm^2^ (SD 0.98) (results not shown). The prevalence of type III (hooked) acromion was 15.8% (134/850), lateral acromial spurs was 11.1% (94/850), medial acromial spurs was 6.6% (56/850), acromioclavicular OA was 12.0% (102/850), and Bankart/Hill-Sachs lesions was 7.1% (60/850). Patients aged 50–63 years had a higher prevalence of calcification, type III (hooked) acromion, lateral spurs, and—particularly among male patients—acromioclavicular OA and Bankart/Hills Sachs lesions. Female patients had a higher prevalence of calcification than male patients. Regarding the architectural measures, we found a mean acromial tilt of 32.9° (SD 5.5°). The mean acromial tilt was 1.6° higher among female patients than among male patients (*p* < 0.01). For males, the mean acromial tilt was 32.1° (SD 5.6) and for females 33.7 (SD 5.4). The mean acromion index was 0.66 (SD 0.09) with no sex difference (*p* =  < 0.01). For lateral acromial angle, the mean angle was 84.4° (SD 7.9°) with a trend towards a higher angle among male patients (1.3°). For males the mean acromial angle was 85.1° (SD 7.4°) and for females 83.8° (SD 8.2°) (appendix 2).

**Table 2:** Prevalence of radiographic findings in orthopaedic patients examined on suspicion of subacromial impingement syndrome according to sex and age group (*N* = 850)

	All patients, *n* = 850		Male patients, *n* = 393				Female patients, *n* = 457			
			18–49 years		50–63 years		18–49 years		50–63 years	
			*n* = 207		*n* = 186		*n* = 244		*n* = 213	
	*n*	%	*n*	%	*n*	%	*n*	%	*n*	%
Calcification										
No	643	75.6	179	86.5	150	80.7	177	72.5	137	64.3
Yes	207	24.4	28	13.5	36	19.3	67	27.5	76	35.7
Calcification area										
No calcification	643	75.6	179	86.5	150	80.7	177	72.5	137	64.3
0.1–0.2 cm^2^	60	7.1	5	2.4	9	4.8	16	6.6	30	14.1
> 0.2–0.6 cm^2^	73	8.6	11	5.3	12	6.4	27	11.1	23	10.8
> 0.6–7.6 cm^2^	74	8.7	12	5.8	15	8.1	24	9.8	23	10.8
Molé classfication										
No calcification	643	75.7	179	86.5	150	80.7	177	72.5	137	64.3
Types A + B	45	5.3	9	4.4	8	4.3	16	6.6	12	5.6
Type C	45	5.3	4	1.9	6	3.2	6	2.5	8	3.8
Type D	138	16.2	15	7.2	22	11.8	45	18.4	56	26.3
Acromial type										
I Flat	109	12.8	28	13.5	23	12.4	39	16.0	19	8.9
II Curved	525	61.8	124	59.9	106	56.9	153	62.7	142	66.7
III Hooked	134	15.8	33	16.0	34	18.3	32	13.1	35	16.4
Missing ^†^	82	9.6	22	10.6	23	12.4	20	8.2	17	8.0
Lateral spur										
No lateral spur	756	88.9	193	93.2	159	85.5	224	91.8	180	84.5
Heal-type	36	4.2	5	0.5	12	6.5	10	4.1	9	4.2
Traction-type	40	4.7	8	3.9	11	5.9	5	2.0	16	7.5
Birdbeak-type	18	2.1	1	0.48	4	2.1	5	2.0	8	3.8
Medial spur										
No medial spur	794	93.4	198	95.7	170	91.4	225	92.2	201	94.4
Traction-type	27	3.2	5	2.4	5	2.7	12	4.9	5	2.3
Birdbeak-type	29	3.4	4	1.9	11	5.9	7	2.9	7	3.3
Acromioclavicular OA										
No	748	88.0	190	91.8	146	78.5	224	91.8	188	88.3
Yes	102	12.0	17	8.2	40	21.5	20	8.2	25	11.7
Bankart/Hill-Sachs-lesion										
No	790	92.9	202	97.6	168	90.3	228	93.4	192	90.1
Yes	60	7.1	5	2.4	18	9.7	16	6.6	21	9.9
Acromial tilt										
13.6–30°	227	26.7	63	30.4	60	32.3	52	21.3	52	24.4
> 30–35°	272	32.0	67	32.4	57	30.6	82	33.6	66	31.0
> 35–50.7°	259	30.5	50	24.2	48	25.8	89	36.5	72	33.8
Missing ^†^	92	10.8	27	13.0	21	11.3	21	8.6	23	10.8
Acromion index										
0.23–0.6	167	19.7	31	15.0	42	22.6	49	20.1	45	21.1
> 0.6–0.7	388	45.6	101	45.8	80	43.0	110	45.1	97	45.5
> 0.7–0.96	288	33.9	71	34.3	64	34.4	83	34.0	70	32.9
Missing ^†^	7	0.8	4	1.9	0	0.0	2	0.8	1	0.5
Lateral acromial angle										
50.3–80°	225	26.5	38	18.4	46	24.7	73	29.9	68	31.9
> 80–90°	431	50.7	115	55.5	100	53.8	115	47.1	101	47.4
> 90–121°	182	21.4	49	23.7	39	21.0	51	20.9	43	20.2
Missing ^†^	12	1.4	5	2.4	1	0.5	5	2.1	1	0.5

^†^ Due to insufficient or missing projection. OA osteoarthritis

Table 3 presents the inter- and intrarater reliability of the radiographic findings. For the presence of calcification, the inter- and intrarater reliability was almost perfect (Kappa values 0.85 and 0.89), while substantial reliabilities were found for acromial type (Kappa values 0.63 and 0.66), calcification density (Kappa intrarater value 0.61), and calcification dissemination (Kappa intrarater value 0.65). Moderate reliabilities were found for lateral spur (Kappa values 0.51 and 0.51), lateral spur type (Kappa values 0.49 and 0.44), acromioclavicular OA (Kappa interrater value 0.41), calcification density (Kappa interrater value 0.57), calcification homogeneity (Kappa intrarater value 0.44), calcification circumscription (Kappa interrater value 0.42), calcification insertion zone (Kappa intrarater value 0.56), and Moléʼs calcification classification (Kappa intrarater value 0.43).

**Table 3:** Inter- and intrarater reliability of radiographic findings

		Interrater			Intrarater ^‡^		
	*N*	Expected agreement %	Observed agreement %	Kappa	Expected agreement %	Observed agreement %	Kappa
Calcification presence	200	52.9	93.0	0.85	53.1	95.0	0.89
Calcification qualities							
Density	69/70^†^	56.0	81.2	0.57	55.8	82.9	0.61
Homogeneity	69/70^†^	48.5	68.1	0.38	49.1	71.4	0.44
Circumscription	69/70^†^	49.8	71.0	0.42	49.8	64.3	0.29
Dissemination	69/70^†^	80.1	90.0	0.34	75.5	91.4	0.65
Insertion zone	69/70^†^	52.1	58.0	0.12	51.0	78.6	0.56
Acromial type	200	74.8	90.7	0.63^*^	73.6	90.9	0.66^*^
Lateral spur presence	200	80.8	90.5	0.51	80.8	90.5	0.51
Lateral spur type	200	84.1	91.8	0.49*	85.1	91.7	0.44*
Medial spur presence	200	90.0	92.5	0.25	89.1	93.5	0.40
Acromioclavicular osteoarthritis	200	79.6	88.0	0.41	82.4	87.5	0.29
Molés calcification classification	69	61.8	60.5	0.03^*^	59.1	59.4	0.43^*^

^*^ Weighted kappa

^†^ Number in inter-/intrarater analyses (one calcification was only seen by one of the raters)

^‡^ Common intrarater value (test–retest performed by any of the two raters)

**Fig. 9:**
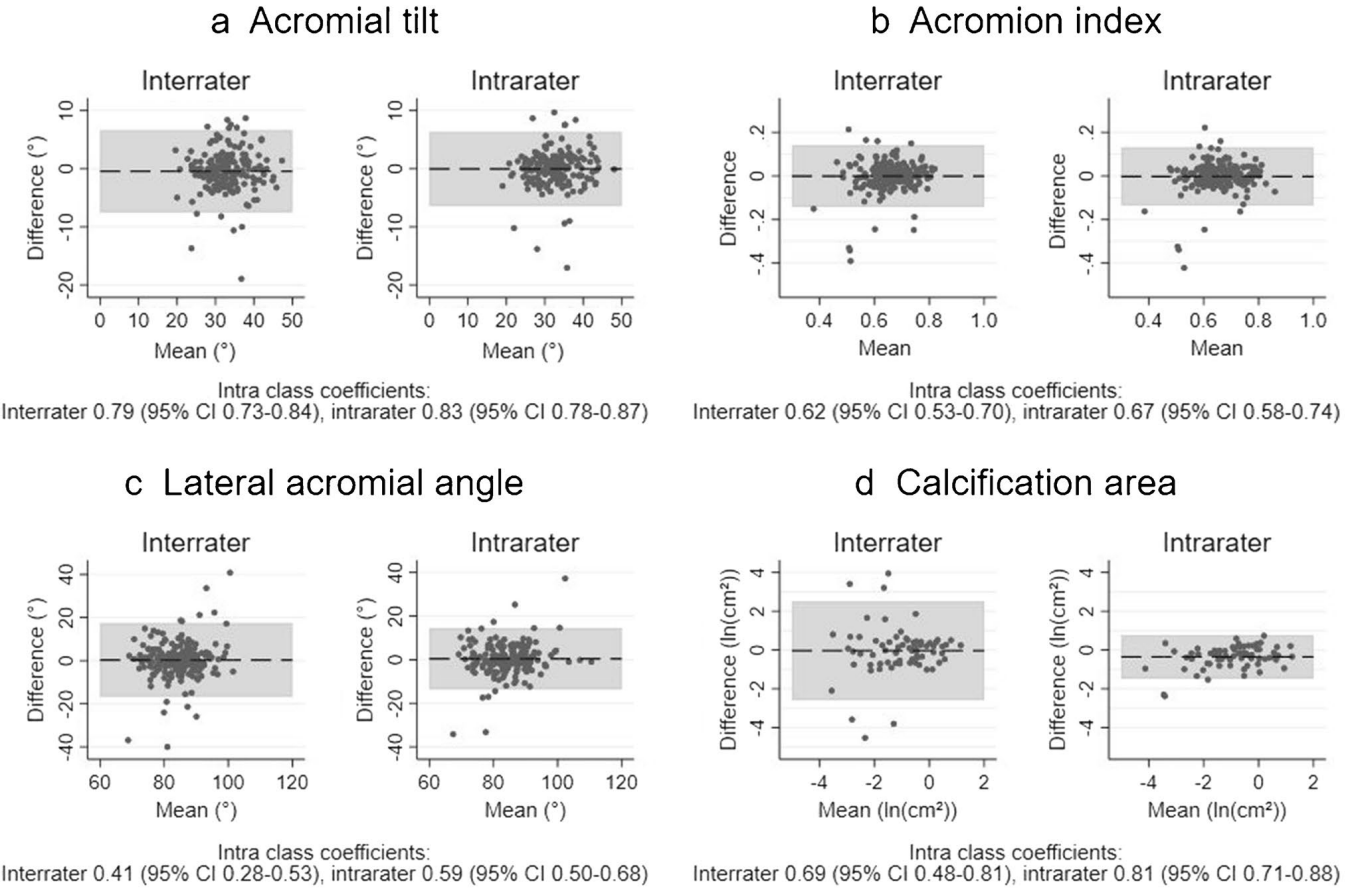
Architectural measures and calcification size: Bland–Altman plots with limits of agreement and intraclass correlation coefficients

Figure 9 presents the Bland–Altman plots for architectural measures and calcification area. Overall, we found no systematic difference between the two evaluators in relation to size of measures. Inter- and intrarater ICC_agreement_ for acromial tilt was excellent (ICC_agreement_ 0.79 and 0.83), ICC_agreement_ for acromion index was fair to good (ICC_agreement_ 0.62 and 0.67), ICC_agreement_ for lateral acromial angle was fair to good (ICC_agreement_ 0.41 and 0.59), while excellent ICC_agreement_ (ICC_agreement_ 0.69 and 0.81) was found for calcification area.

## Discussion

Among patients referred to an orthopaedic department on suspicion of SIS, only 25% of routine radiographic examinations complied with current recommendations of three projections. Calcification, Bigliani type III (hooked) acromion, and acromioclavicular osteoarthritis were prevalent findings among patients seen in orthopaedic departments on suspicion of SIS with prevalence between 12.0 and 24.4%. Spurs and Bankart/Hill-Sachs lesions were less common (6.6–11.1%). Inter- and intrarater Kappa values for most radiographic findings ranged between 0.40 and 0.89; highest values for the presence of calcification (0.85 and 0.89) and acromion type (0.63 and 0.66). The inter- and intrarater intraclass coefficients ranged between 0.41 and 0.83; highest values for acromial tilt (0.79 and 0.83) and calcification area (0.69 and 0.81). The results are generally in agreement with our hypothesis.

### Strengths and limitations

Inaccessible radiographs (*N* = 189) were due to technical reasons and had no association to the actual radiographic findings. Low quality of radiographs could have influenced the prevalence of findings, which might be underestimated due to missing projections. This is not different in previous studies with similar prevalence, and we have no reason to believe that the quality in our study differs from others. Mutual calibration of the evaluators and the use of a detailed manual have strengthened the reliability of findings. In classification of calcifications, it is important to keep in mind the somewhat unclear differentiation of subclasses, which is an information bias in present as well as previous studies.

Only 25% of the radiographic examinations included all the recommended projections. We decided to include the examinations with less than 3 views available to reflect the clinical setting, where the radiograph was taken. Although we use a detailed evaluation manual aimed at standardising the evaluations to the largest possible extent and only two evaluators were involved, the low reliabilities for some radiographic findings may call for further standardisation.

The coverage of the project database was only around one third of the eligible patients according to the Danish National Patient Register, but missing registration in the project database was primarily explained by practical issues, in particular periods with high workload in outpatient clinics and without project secretaries/nurses in the individual participating departments, which diminishes the risk of selection bias. Non-response analysis showed an even age- and sex distribution among non-included.

### Prevalence of radiographic findings

We found a prevalence of calcifications (24.4%) in level with some of the earlier reported prevalence among patients with shoulder pain [58, 59] as well as in level with prevalence in both a population of supermarket cashiers and their reference group of customers [20]. Previously reported prevalence of calcifications that differs from our findings have also been reported. Studies of patients in general practice patients with a first-time episode of shoulder pain, report prevalence estimates of 2.7% [10] to 14.8% [8, 9]. Lower prevalence in patients reporting first time to general practitioner than in our hospital setting is however not surprising as patients referred to hospitals include more severe cases. We found a higher prevalence among female patients and older patients which is supported by a previous study [6]. The higher prevalence in female and older patients could be expected since calcific tendonitis is a multifactorial disease with, among other, genetic, hormonal and degenerative factors suspected to play a role in the pathogenesis [60, 61].

The frequency of a hooked acromion (Type III) in our study (12%) is supported by other studies [31, 44]. We found a four times lowered prevalence of lateral acromial spurs in our study than previously reported [39]. However, the previously reported high prevalence stems from a population of significantly older patients (min–max 45–79 years, mean 59.6 years) with a higher fraction of male patients (52%) which could explain the difference in findings.

### Reliability of radiographic findings

The reliability of classifications of calcifications is in accordance with previous studies that have shown light to moderate inter- and intrarater reliabilities [18, 26, 27]. We reached a higher interrater reliability of acromial type according to Bigliani than previously reported [7, 62], which suggest that the use of a detailed manual strengthens evaluation of acromial type. Previously reported interrater agreement of acromial tilt has been reported as fair to good [24]. This study strengthens the previous findings of acromial tilt as a reliable measure. In our study, fair inter- and intrarater agreement was reached for the presence of a lateral spur. Previously, fair to moderate interrater reliabilities and moderate intrarater reliability have been reported [39, 62].

### Generalisability and perspectives

In our study, the inclusion of patients was closely related to the clinical settings we intended to describe, in the sense that we included patients referred for orthopaedic evaluation on suspicion of SIS. In Denmark, the health care system is public, and treatment is paid via taxes. The socioeconomic difference in treatment of patients is, to some extent, minimised by this free access to treatment. Therefore, we think our results can be generalised to other countries, with a similar access to treatment or where health insurance coverage is high.

This study described the radiographic findings in a large group of patients with suspicion of SIS.

For radiographic findings to be relevant with respect to diagnosis, choice of treatment, and inference of prognosis, the findings must play a role for the patients’ symptoms. Association between specific radiographic findings and shoulder symptoms and disability in patients with suspected SIS is warranted.

The quality of the radiographs, with only 25% including the recommended projections, call for further standardisation of the examination. In conclusion of the findings, calcification, Bigliani type III (hooked) acromion, and acromioclavicular OA were prevalent findings among patients seen in orthopaedic departments on suspicion of SIS, while spurs and Bankart/Hill-Sachs lesions were less common. Optimal reliabilities were found for the presence calcification, calcification area, and acromial tilt, while acromioclavicular OA, calcification qualities, and calcification classification showed suboptimal reliability.

## Appendix 1

See Table

**Table 4:** Comparison of four systems for classification of subacromial calcifications based on calcification qualities

	Density		Homogeneity		Circumscription		Dissemination		Insertion zone	
	Dense	Transparent	Homogeneous	Inhomogeneous	Well-defined	Ill-defined	No	Yes	No	Yes
Gärtner										
1	X		IR	IR	X		IR	IR	IR	IR
2a	X		IR	IR		X	IR	IR	IR	IR
2b		X	IR	IR	X		IR	IR	IR	IR
3		X	IR	IR		X	IR	IR	IR	IR
Patte										
1	IR	IR	X		X		X		IR	IR
2	IR	IR		X		X		X	IR	IR
De Palma										
1	IR	IR		X		X	IR	IR	IR	IR
2	IR	IR	X		X		IR	IR	IR	IR
Molé										
A	X		X		X		IR	IR	X	
B	X			X	X		IR	IR	X	
C		X		X		X	IR	IR	X	
D							IR	IR		X

IR: Irrelevant

4

## Appendix 2

See Table

**Table 5:** Architectural measures in orthopaedic patients examined on suspicion of subacromial impingement syndrome according to sex according to sex

	Architectural measurements		
Measure (male/female)	Total (*N* = 850)	Male (*N* = 393)	Female (*N* = 457)
Acromial tilt (413/345)	32.9° (SD 5.5)	32.1 (SD 5.6)	33.7 (SD 5.4)
Acromion index (454/389)	0.66 (SD 0.09)	0.67 (SD 0.09)	0.66 (SD 0.09)
Lateral acromial angle (451/387)	84.4° (SD 7.9°)	85.1 (SD 7.4)	(SD 8.2)

5
